# A whole blood monokine-based reporter assay provides a sensitive and robust measurement of the antigen-specific T cell response

**DOI:** 10.1186/1479-5876-9-143

**Published:** 2011-08-26

**Authors:** Aron Chakera, Sophia C Bennett, Richard J Cornall

**Affiliations:** 1 Nuffield Department of Medicine, Old Road Campus, University of Oxford, Roosevelt Drive, Oxford, OX3 7BN, UK

## Abstract

**Background:**

The ability to measure T-cell responses to antigens is proving critical in the field of vaccine development and for understanding immunity to pathogens, allergens and self-antigens. Although a variety of technologies exist for this purpose IFNγ-ELISpot assays are widely used because of their sensitivity and simplicity. However, ELISpot assays cannot be performed on whole blood, and require relatively large volumes of blood to yield sufficient numbers of peripheral blood mononuclear cells. To address these deficiencies, we describe an assay that measures antigen-specific T cell responses through changes in monokine gene transcription. The biological amplification of the IFNγ signal generated by this assay provides sensitivity comparable to ELISpot, but with the advantage that responses can be quantified using small volumes of whole blood.

**Methods:**

Whole blood or peripheral blood mononuclear cells (PBMCs) from healthy controls and immunosuppressed recipients of solid organ transplants were incubated with peptide pools covering viral and control antigens or mitogen for 20 hours. Total RNA was extracted and reverse transcribed before amplification in a TaqMan qPCR reaction using primers and probes specific for MIG (CXCL9), IP-10 (CXCL10) and HPRT. The induction of MIG and IP-10 in response to stimuli was analysed and the results were compared with those obtained by ELISpot.

**Results:**

Antigen-specific T cell responses can be measured through the induction of MIG or IP-10 gene expression in PBMCs or whole blood with results comparable to those achieved in ELISpot assays. The biological amplification generated by IFNγ-R signaling allows responses to be detected in as little as 25 μL of whole blood and enables the assay to retain sensitivity despite storage of samples for up to 48 hours prior to processing.

**Conclusions:**

A monokine-based reporter assay provides a sensitive measure of antigen-specific T cell activation. Assays can be performed on small volumes of whole blood and remain accurate despite delays in processing. This assay may be a useful tool for studying T cell responses, particularly when samples are limited in quantity or when storage or transportation is required before processing.

## Background

Analysis of T-cell responses to antigen has become central to our understanding of immunity against pathogens [1] and for vaccine development [2]. Over the years, many methods have been developed to assess antigen-specific T cells, progressing from limiting dilution and ^51^Cr release assays to techniques that measure cytokine production and changes in T-cell phenotype upon activation [3,4].

Activation of T-cells by antigen leads to the production of cytokines, which in turn mediate downstream effector responses [5]. Of the cytokines secreted, IFNγ is one of the best studied, having been shown to be a robust marker of T cell activation with levels correlating with the efficacy of the immune response [6,7]. As a result, the detection of intracellular or secreted IFNγ has become an accepted surrogate for functional immune responses to antigen in many circumstances. Of the techniques available to measure IFNγ, ELISpot analyses are one of the most widely used due to their low cost, reliability and high sensitivity for detecting low numbers of antigen-specific cells [8]. However, these assays require purified PBMCs and relatively large volumes of blood to perform.

**Figure 1:**
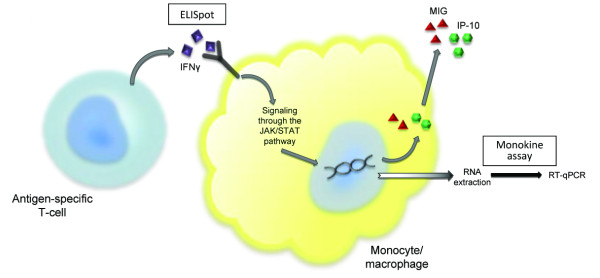
**Antigen activated T cells and the monokine assay**. In contrast to ELISpot assays which capture and measure IFNγ produced by activated T cells, the monokine assay measures the downstream effects of IFNγ on mononuclear cells benefiting from the biological amplification of the signalling.

IFNγ secreted by antigen-activated T-cells binds to interferon-gamma receptors present on several cell types, including those of monocyte/macrophage lineage [9]. Binding causes a conformational change in the receptor and activation of JAK/STAT second messenger pathways [10], which in humans leads to the transcription of a cluster of genes present on chromosome 4 (4q21) including monokine induced by interferon gamma (MIG or CXCL9) and interferon gamma-induced protein of 10 kDa (IP-10 or CXCL10) [11] (Figure 1).

MIG and IP-10 are inflammatory chemokines predominantly released by monocytes following stimulation by IFNγ [12,13], which act as part of a feedback loop binding to the chemokine receptor CXCR3 expressed by activated T lymphocytes [14]. As only minimal transcription of these genes occurs in the basal state [15], the biological amplification of the IFNγ signal by the second messenger signaling pathways provides an opportunity to improve detection when compared with direct measurement of IFNγ [11], however previous experiments have primarily focused on intracellular detection of MIG at the protein level in CD14^+ ^cells via flow cytometry [11,16]. A report demonstrating the detection of MIG at the RNA level in association with T-cell responses to a malaria vaccine supports the idea that this could be used as a bioassay of antigen-specific responses [16], but the potential utility of a monokine-based detection system has not been evaluated systematically.

Therefore, we wanted to know whether an assay measuring the induction of MIG and IP-10 mRNA could be a sensitive and convenient alternative to ELISpot assays or other techniques based on the use of antibody reagents and/or flow cytometry. To this end, we have developed a protocol using qPCR for MIG and IP-10 to quantify antigen-specific T cell responses that can be performed on whole blood or with PBMCs. We show this assay remains sensitive when tested with small volumes of blood, despite delays of up to 48 hours before sample processing and correlates well with the results of IFNγ-ELISpot assays.

## Methods

### Subject recruitment and collection of samples

Patients attending the Oxford Kidney and Transplant Units and healthy controls recruited through advertisement were invited to donate whole blood following written informed consent. Ethical approval for the study was granted by the Berkshire Research Ethics Committee (REC reference 08/H0607/50). Whole blood was collected directly into anticoagulated sodium heparin tubes (BD, Oxford, UK).

### Isolation of PBMCs

Cell separation tubes (Sigma, Gillingham, UK) were used to isolate peripheral blood mononuclear cells (PBMCs) from whole blood by density gradient centrifugation. PBMCs were washed twice with sterile PBS (Fisher Scientific, Loughborough, UK) then counted before being re-suspended in RPMI 1640 media, supplemented with 10% Fetal Calf Serum, 1% L-Glutamine, 1% Penicillin-streptomycin solution, 1% Sodium Pyruvate (all from Sigma), 1% HEPES buffer (GibcoBRL, Scotland, UK), and 0.05 mM 2-Mercaptoethanol (Invitrogen, Paisley, UK).

### Preparation of peptides and reagents

Peptide pools (15 amino acids in length with overlaps of 11 amino acids) comprising the immunodominant EBV antigens BZLF1 and EBNA1, and the CMV-specific antigens pp65, UL-40, IE1 and IE2 were purchased from JPT (Berlin, Germany). Control peptide pools derived from human alpha-actin-1 (ACTs) protein (negative control) and CEF (CMV, EBV and Influenza) peptides (positive control) were also obtained from JPT (Berlin, German). All peptides were stored at -20°C as lyophilised powders until used. Peptide pools were dissolved in DMSO (Sigma) and diluted with Dulbecco's PBS (GibcoBRL) according to the manufacturer's instructions. The lymphocyte mitogens, Concanavalin A (ConA) and Phytohemagglutinin (PHA) and the calcineurin inhibitor tacrolimus (FK506) were purchased from Sigma (Gillingham, UK). Recombinant human IFNγ was obtained from Peprotech (London, UK).

### IFNγ-ELISpot assay

96-well PVDF plates (Millipore, Watford, UK) were coated with 100 μl of IFNγ monoclonal antibody at 15 μg/ml (clone 1-D1K), (MAbTech, Stockholm, Sweden) and incubated at 4°C overnight. The next day, plates were washed 5 times with PBS (Fisher Scientific) then incubated for 2 hours at 37°C with 200 μl/well of complete media. Peptide pools were added in triplicate to each plate at a final concentration of 1 μg/ml. PHA and ConA were used at final concentrations of 5 μg/ml, also in triplicate. 2 × 10^5 ^PBMCs were added to each well and incubated for 18-20 hours at 37°C in 5% CO_2_. At the end of the incubation period, plates were washed 5 times with PBS/0.05% Tween 20 (Sigma) then left at room temperature for 2 hours after the addition of 100 μl of 1 μg/ml biotinylated IFNγ antibody (clone 7-B6-1) (MAbTech). After washing 5 times 100 μl (1 μg/ml) Streptavidin-ALP (MAbTech) was added for 1 hour at room temperature, then plates were developed with 100 μl/well of 0.22 μm filtered BCIP/NBT (Pierce, Cramlington, UK). Development continued until spots appeared in the positive control wells. Spots were counted using an automated plate reader, AID ELISpot (AID, Strassberg, Germany), with AID ELISpot software Version 3.5, (CADAMA Medical Ltd, Stourbridge, UK).

### Monokine Assay

PBMCs or whole blood were aliquoted into 96-well round-bottom plates (Greiner, Gloucestershire, UK) before the addition of peptide pools, mitogen or recombinant IFNγ (all in triplicate). Plates were incubated for 18-20 hours at 37°C, in 5% CO_2 _before RNA extraction using an RNeasy Mini Kit (PBMCs) or QIAamp RNA Blood Mini Kit (whole blood) according to the manufacturer's instructions. Extracted RNA was reverse transcribed using Superscript First-Strand Synthesis System for RT-PCR (Invitrogen, Paisley, UK). cDNA quantification was performed in a 10 μl reaction mix using Taqman Fast Universal Mastermix and the Taqman Gene Expression Assay (Applied Biosystems, Warrington, UK). The Taqman Gene Expression assays included primer and probe combinations for MIG (CXCL9) (Assay ID Hs00171065_m1), IP-10 (CXCL10) (Assay ID Hs00171042_m1) and the housekeeping gene hypoxanthine phosphoribosyltransferase 1 (HPRT) (Assay ID Hs99999909_m1). qPCR was performed on a StepOnePlus Real-Time PCR System (Applied Biosystems). Experimental triplicates were carried through each step of the protocol. qPCR data are expressed as fold induction (RQ value) of MIG or IP-10 normalized to expression of the housekeeping gene HPRT. RQ is a function of the C_t _value, where C_t _is the number of PCR cycles required to produce a sustainable amplification in the fluorescent signal above the background threshold. ΔC_t _is the difference between the C_t _value for the housekeeping gene and the C_t _value for the gene of interest e.g. MIG. ΔΔC_t _is the ΔC_t _value of the stimulated sample subtracted from the ΔC_t _value of the reference (negative control). RQ is calculated as 2^-ΔΔCT^.

The efficiency (or E value) of the quantitative PCR reactions was calculated from a standard curve of MIG and IP-10 induction following stimulation with increasing concentrations of recombinant IFNγ. The average E values for whole blood and PBMCs were 0.997 and 0.998 respectively (with no difference between MIG and IP-10). The human acute monocytic leukaemia cell line THP-1 (ATCC, Middlesex, UK) was used for *in vitro *analyses, and as an external control. To facilitate inter and intra-patient comparison RQ values were routinely normalized to the mean fold induction of MIG and IP-10 in response to the negative control.

### Data analysis

Statistical analyses were performed with GraphPad PRISM Version 5 (GraphPad Software Inc, California, USA). Differences between groups were analysed by one-way analysis of variance (ANOVA) or independent t-tests. Normality was assessed using the D'Agostino-Pearson omnibus test. Statistical significance was defined as p < 0.05. Data from monokine assay are presented as the mean and 95% confidence interval calculated from mean, maximum and minimum RQ values from experimental triplicates and ELISpot data as the mean ± SEM from triplicate wells.

## Results

### Recombinant IFNγ induced MIG and IP-10 in culture

**Figure 2:**
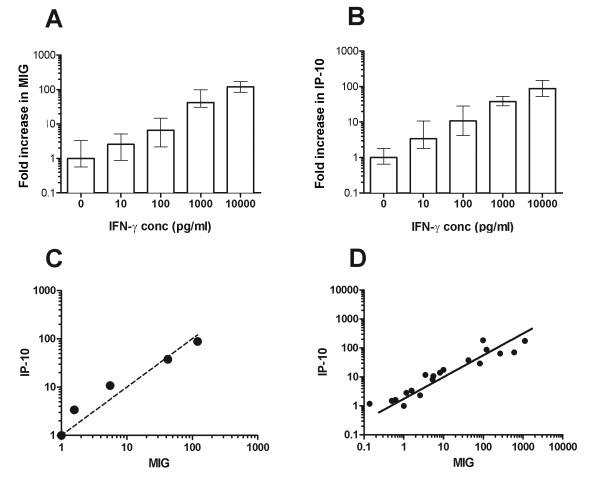
**Induction of MIG (Panel A) and IP-10 (Panel B)**. PBMCs from healthy donors were stimulated with increasing concentrations of recombinant human IFNγ. The correlation between dose of IFNγ and MIG expression (A) was r^2 ^= 0.94, and with IP-10 expression (B) was r^2 ^= 0.90. Similar results were obtained using the mononuclear THP-1 cell line (data not shown). Columns show the mean RQ value and 95% confidence intervals calculated from triplicate wells. (C) The relationship between the induction of MIG and IP-10 following the addition of recombinant IFNγ (from A&B above), with reference to the induction of MIG (dashed line). (D) The correlation between MIG and IP-10 induction in 23 subjects following stimulation with a variety of peptides (r^2 ^= 0.9885).

To discover whether the induction of MIG and IP-10 mRNA could be used as a robust measurement of antigen-specific T cell activation and IFNγ release, we initially studied the effects of recombinant IFNγ added directly to freshly isolated PBMCs or control THP-1cells. 2 × 10^5 ^cells/well were incubated for 16 hours with medium alone or 10-10,000 pg/ml IFNγ. Experimental triplicates were carried through each step of the assay and all assays were performed a minimum of five times. Up-regulation of both MIG and IP-10 was detectable at all concentrations of IFNγ tested compared with unstimulated controls, although at the lowest concentration (10 pg/ml) only IP-10 induction was significantly increased above baseline (fold induction, RQ = 3.4, p = 0.013 for IP-10 and RQ = 2.6, p = 0.16 for MIG), suggesting that IP-10 may be more sensitive for the detection of low concentrations of IFNγ (Figure 2A-C). To further explore the relationship between the induction of MIG and IP-10, this time in the context of antigen-activated T cells, we analysed the changes in gene transcription in samples from 23 different individuals stimulated with a variety of peptides (Figure 2D). The correlation between the induction of MIG and IP-10 in these samples was highly significant r^2 ^= 0.9885.

### Antigen-specific responses can be measured in whole blood or PBMC

**Figure 3:**
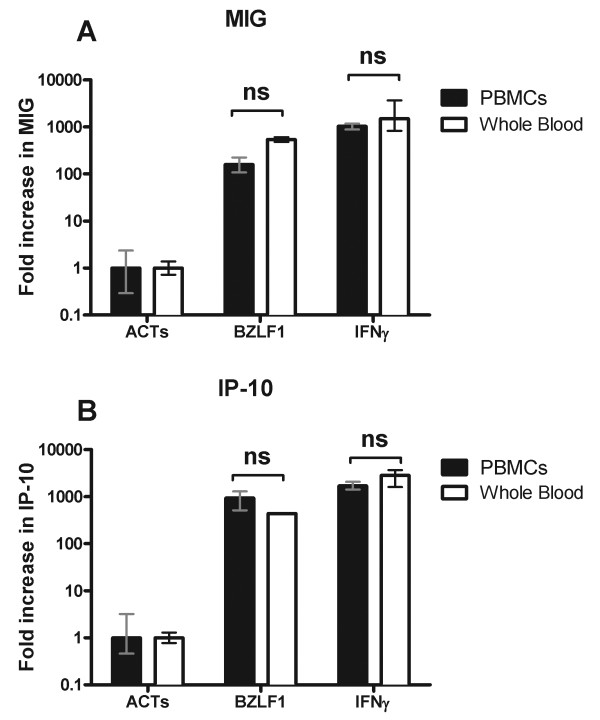
**Comparison of MIG and IP-10 induction in whole blood vs. PBMCs**. Whole blood (100 μl) clear bars or PBMCs (2 × 10^5^) filled bars were incubated with peptides spanning the EBV antigen BZLF1, human actin (ACTs) or recombinant IFNγ (10,000pg/ml) for 18-20 hours before lysis and RNA harvesting. The induction of MIG (Panel A) and IP-10 (Panel B) gene expression are shown normalized to the negative control (ACTs). Columns show the mean RQ value and 95% confidence intervals calculated from triplicate wells. Results are representative of five individual experiments.

A significant limitation of ELISpot assays is the requirement to isolate PBMCs. Because the monokine assay measures the downstream effects of IFNγ on gene transcription, we questioned whether it would be possible to perform these assays using whole blood. To test whether the induction of IP-10 and MIG could provide a sensitive measure of IFNγ production by antigen activated T cells *ex vivo*, we incubated whole blood (100 μl) or PBMCs (2 × 10^5^) from individuals with known responsiveness to the EBV antigen BZLF1 by ELISpot, with overlapping peptide pools covering BZLF1 or human actin (ACTs). The addition of 10,000 pg/ml recombinant IFNγ is shown for comparison (Figure 3). (100 μl of whole blood was chosen on the basis that the average yield of PBMCs from the donors was ~2 × 10^6^/ml). This experiment showed equivalent (p > 0.05) up-regulation of both IP-10 and MIG in response to antigen compared to the negative control in both PBMCs (p = 0.0003 for IP-10 and p = 0.0008 for MIG) and whole blood (p < 0.0001 for IP-10 and MIG).

### Effects of sample storage on assay reproducibility

**Figure 4:**
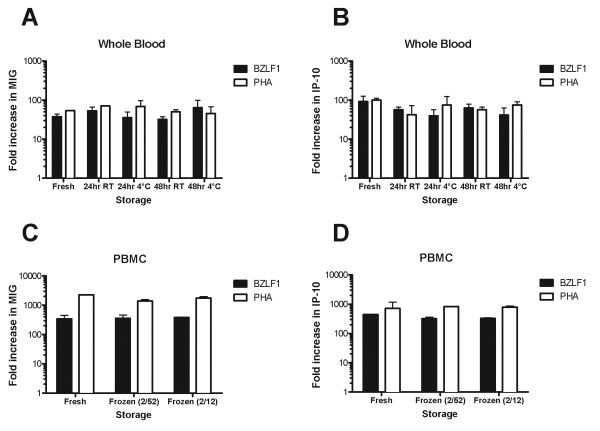
**Effects of storage on assay sensitivity**. Whole blood (Panels A & B) or PBMCs (Panels C & D) were stored for variable periods of time at room temperature (RT), 4°C (whole blood) or -80°C (PBMCs) before stimulation with BZLF1 or PHA for 18 hours followed by RNA extraction. Induction of MIG (Panels A&C) and IP-10 (Panels B&D) mRNA transcripts are shown normalized to the negative control (ACTs). Columns show the mean RQ value and 95% confidence intervals calculated from triplicate wells. Results are representative of three individual experiments.

A further practical concern in planning and executing successful experiments in the clinical setting are delays before samples are processed, especially when they are collected in the field. Therefore, we asked next to what extent the signal from the monokine assay would be able to survive intact without rapid processing. Blood from healthy controls with known responsiveness to BZLF1 was divided for processing as either whole blood (100 μL) or PBMCs (2 × 10^5^). Whole blood samples were incubated with antigen immediately or after 24 or 48 hours storage at room temperature or 4°C. PBMCs were assessed fresh or after 2 weeks or 2 months storage in liquid nitrogen (Figure 4). These experiments showed that antigen-specific responses in whole blood did not significantly degrade despite up to 48 hours at either room temperature or 4°C (Figure 4A and 4B). Although careful cryopreservation of whole blood samples has previously been reported to maintain lymphocyte viability and functionality [17], this resulted in almost complete loss of the antigen-specific response, and only minor preservation of the response to mitogen (data not shown). Therefore reliable cryopreservation depends on the isolation of PBMCs, which can be stored for up to 2 months without any loss in sensitivity of the assay (Figure 4C and 4D).

### Sensitivity with reduced numbers of PBMCs or volume of whole blood

**Figure 5:**
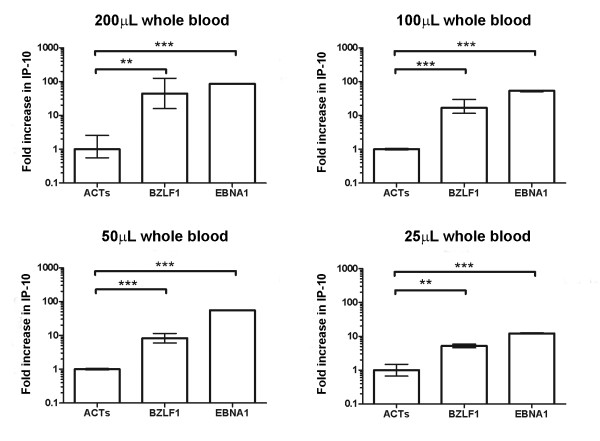
**Reducing the volume of whole blood**. The sensitivity of the assay to detect antigen-specific T cell responses despite a reduction in sample volume was assessed using healthy controls with known responsiveness to the immunodominant EBV antigens BZLF1 and EBNA1. A statistically significant increase in both MIG and IP-10 (data for IP-10 is shown) could be detected following stimulations performed in as little as 25 μL of whole blood. Columns show the mean RQ value and 95% confidence intervals calculated from triplicate wells (** = p < 0.01, *** = p < 0.001).

Encouraged by the whole blood response, we questioned whether the biological amplification of the IFNγ signal in monocytes might permit the detection of meaningful responses with lower PBMC numbers or smaller volumes of whole blood. Decreasing the number of PBMCs resulted in a predictable reduction in the magnitude of the response to BZLF1; however responses still remained detectable above background when as little as 25 μl of whole blood (Figure 5) or 50,000 PBMCs were used (Additional file 1). Preservation of the response to exogenous IFNγ despite a reduction in PBMC numbers suggests that antigen-specific T-cell numbers, rather than mononuclear cell numbers limit the response (Additional file 1).

### Comparison of monokine and IFNγ-ELISpot assays

**Figure 6:**
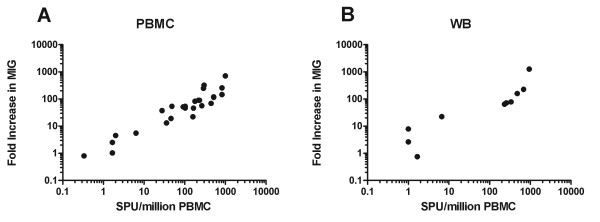
**Correlation between monokine and ELISpot assays**. A) Responses to the CEF pool, EBV and CMV peptides and PHA were analysed in PBMCs from 25 subjects. The correlation between the induction of MIG gene transcription in the monokine assay (y axis) and number of spot forming units (SFU)/million PBMCs (X axis) was (r^2 ^= 0.592, p < 0.0001). B) Similar analysis as A) comparing MIG induction in whole blood samples with ELISpot from 10 further donations (r^2 ^= 0.67292, p = 0.0037).

As ELISpot assays are the method of choice for measuring antigen-specific T cells in many laboratories, we next assessed whether responses detected using the monokine assay would be comparable to those achieved by ELISpot. In initial experiments we compared responses using PBMCs from 25 subjects plated at 2 × 10^5^/well in triplicate, stimulated with a variety of peptides (CEF pool, BZLF1, EBNA1, UL-40, pp65, IE1 and IE2) or mitogen (PHA). Fold induction of MIG and IP-10 were compared to the number of spot forming units (SPU) seen by ELISpot (Figure 6A). The correlation between MIG induction and SPU/million PBMCs was r^2 ^= 0.592 (p < 0.0001). The correlation between MIG and IP-10 induction was r^2 ^= 0.981.

Having confirmed a highly significant relationship between the results of ELISpot and monokine assay using PBMCs, we proceeded to assess whether a similar relationship might be evident with whole blood. T-cell responses to stimulation with the CEF peptide pool, CMV or EBV peptides as above, were measured in a further 10 samples using 100 μl/well of whole blood for the monokine assay or 2 × 10^5 ^PBMCs/well for the ELISpot assay (Figure 6B). The correlation between the fold increase in MIG in the monokine assay and SPU/million PBMCs in the ELISpot assay was r^2 ^= 0.6729 (p < 0.0037). Except in one case, where low-level induction of MIG and IP-10 was seen when the ELISpot was considered negative (21 fold-induction of MIG vs. 7 SPU/million PBMCs), there was concordance between ELISpot and monokine assay results.

### Effects of immunosuppression

**Figure 7:**
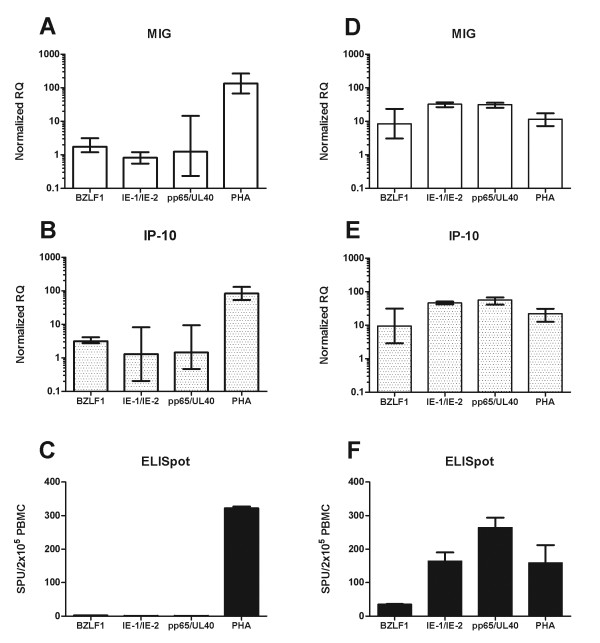
**Comparison of monokine and ELISpot assays in an immunosuppressed patient**. Induction of MIG and IP-10 were compared with the number of spot forming units detected by ELISpot in two patients with CMV viremia. Patient A (Panels A-C) had uncontrolled CMV viremia with persistently elevated viral titres. Patient B (Panels D-F) had resolving viremia (decreasing viral titres). Responses to peptides from human actin (ACTs), EBV (BZLF1), and early (IE-1&IE-2) and late (pp65&UL-40) CMV antigens as well as PHA were measured ELISpot responses are shown as the mean ± SEM from triplicate wells following subtraction of the average number of spots seen in the negative control (ACTs) wells (1 for Patient A and 72 for Patient B). Monokine assay results are presented as the mean RQ value and 95% confidence interval normalized to the negative control (ACTs), calculated from triplicate wells. The high background spot numbers seen for patient B are consistent with widespread immune activation during recovery from viremia.

A clinically important situation for the detection of antigen-specific responses is in the immunocompromised host. Therefore, we wanted to test the monokine assay in this setting, particularly in light of the potentially modulating effects of immunosuppressant medications on JAK/STAT signaling [18]. Accordingly, we analysed responses to peptides or mitogen in two renal transplant recipients, one with uncontrolled CMV viremia and one with resolving CMV viremia (Figures 7A-C and 7D-E respectively). 2 × 10^5 ^PBMCs were stimulated (in triplicate), and the number of spot-forming units measured by ELISpot compared with the fold induction of IP-10 and MIG. The average number of spots seen in the negative controls wells was subtracted from each well in the ELISpot assay to account for any background activation while monokine assay results were normalised to the ACTs response, so that net antigen-specific responses are shown. The results of the ELISpot assay were mirrored by the changes seen in MIG and IP-10 gene expression (Figure 7).

**Figure 8:**
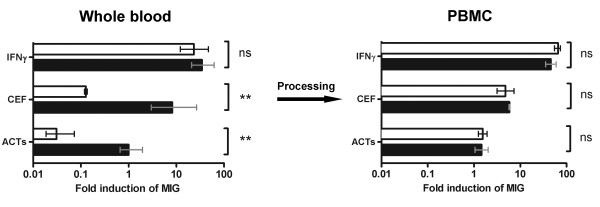
**Comparison of whole blood and PBMC responses in the presence of immunosuppression**. The effect of the calcinuerin inhibitor tacrolimus on T cell responses to antigen was assessed in whole blood (left panel) and PBMCs (right panel). Incubation with 20 ng/ml tacrolimus (clear bars) but not PBS (black bars) significantly inhibited antigen-specific T cell responses in whole blood when compared with PBMCs. Mean RQ values and the 95% confidence interval are shown normalized to the ACTs response for PBS-incubated samples.

Purification of PBMCs from whole blood can lead to their direct activation, as a result of processing [19,20], or indirect activation through loss of the inhibitory effects of therapeutic agents that may be present *in vivo*. As a result, the assessment of antigen-specific responses in whole blood may provide a more clinically meaningful measure of immune function. To test this hypothesis, we next collected whole blood from healthy donors and incubated samples in the presence or absence of tacrolimus (FK506), an immunosuppressive agent commonly used to prevent rejection in recipients of solid organ transplants [21]. Samples were then divided for processing as whole blood or PBMCs, as described above. While the induction of MIG or IP-10 following direct stimulation with recombinant IFNγ was unaffected by incubation with tacrolimus in PBMCs or whole blood, treatment with tacrolimus significantly inhibited T cell responses to antigen in whole blood (p = 0.0046 for the CEF pool) (Figure 8).

## Discussion

The ability to accurately quantify antigen-specific responses has become integral to the study of infectious diseases, vaccine responses, autoimmunity and allergy [4]. While a variety of techniques exist to detect T cells that recognize specific epitopes and to analyse the effects of antigen-induced activation on T cell phenotype, many of these methods require sophisticated equipment and significant expertise to perform. An alternative strategy has been the measurement of cytokines produced by antigen-activated T cells, in particular IFNγ, which has been demonstrated to correlate with functional immunity in a range of clinical settings [22,23].

ELISpot assays have become the standard method to quantify IFNγ production in many laboratories because they are easy to perform, do not require investment in expensive equipment (e.g. flow cytometers) and are sensitive for the detection of even low numbers of antigen-specific cells [24]. However, ELISpot assays have several important limitations: relatively large volumes of blood are required to obtain sufficient PBMCs for analysis; the process of extracting and purifying PBMCs may cause their activation and alter their phenotype [19,20]; assays cannot be re-run unless stored samples of cells are available.

Due to the high burden of infectious diseases in the developing world, much of the research into T-cell responses to pathogens is focussed on areas where there are limited resources for the extraction and purification of PBMCs and where sample volumes may be restricted by patient age or co-morbidity. In these settings an assay that requires only small volumes of blood and which retains sensitivity despite delays in processing would provide significant benefits. In this study we have described a method to assess antigen-specific T cell responses *ex vivo *by measuring the up-regulation of MIG and IP10 gene transcription in mononuclear cells, in response to IFNγ released by activated T cells (Figure 1). We analysed responses to both chemokines as a means of internally validating our results and as expected the correlation between MIG and IP10 induction was high. Interestingly, the induction of IP-10 appears to be a more sensitive marker in the presence of low numbers of antigen-specific T cells, possibly reflecting activation of IP-10 gene transcription by cytokines other than IFNγ (Figure 2) [25].

In contrast with previous studies that have evaluated antigen-specific responses through changes in T-cell cytokines, we have found the monokine assay to have several advantages. The biological amplification of the IFNγ signal in monocytes means that co-stimulatory antibodies are not required [26]; responses remain stable despite delays in processing (Figure 4), or where samples sizes are small (Figure 5) and sensitivity equivalent to ELISpot is achievable [27]. Furthermore, by enabling the measurements to be made on whole blood, preparation time is reduced and results may be more representative of the response *in vivo *[28,29] (Figure 8).

As the assessment of antigen-specific responses in the monokine assay is indirect, one limitation of the assay is its inability to distinguish between a small number of highly activated T cells and a larger number of less activated T cells producing the same amount of IFNγ. Although this necessarily reduces the strength of the relationship between monokine and ELISpot assay results, overall the correlation between these assays is good and significantly, non-responders are clearly identified (Figure 6). Additional benefits of the monkine assay are that by measuring the effects of IFNγ on target cells, rather than the production of IFNγ alone, the monokine assay provides some confirmation of the downstream functionality of the T-cell response and once RNA has been extracted and reverse transcribed, samples can be stored indefinitely.

## Conclusions

The biological amplification of the IFNγ signal generated by the endogenous reporter system described provides a sensitive and accurate way to measure antigen-specific T cell activation. The opportunity to perform these assays on whole blood reduces the time and resource requirements for sample processing and may yield results that are more representative of the *in vivo *situation. In addition, the retention of sensitivity despite delays in processing or where samples are limited in quantity will make this assay a viable alternative to ELISpot in many settings.

## Competing interests

The authors declare that they have no competing interests.

## Authors' contributions

AC, SB and RJC designed the study. AC and SB performed the experiments. AC, SB and RJC wrote the manuscript. All authors have read and approved the final manuscript.

## Supplementary Material

Additional file 1 **Detection of antigen-specific responses with reduced numbers of PBMCs**. Induction of MIG (A) and IP-10 (B) mRNA in response to stimulation with BZLF1 peptides or recombinant IFNγ in decreasing numbers of PBMCs is shown. (Results are presented as RQ values normalized to the negative control ACTs). Significant induction of MIG and IP-10 can be detected with as few as 1 × 10^5 ^cells/well for MIG or 0.5 × 10^5 ^cells/well for IP-10. Click here for file
