# Supplementary figures and images for: A whole blood monokine-based reporter assay provides a sensitive and robust measurement of the antigen-specific T cell response
Source: J Transl Med. 2011 Aug 26;9:143. doi: 10.1186/1479-5876-9-143 (PMC3179727; doi:10.1186/1479-5876-9-143)

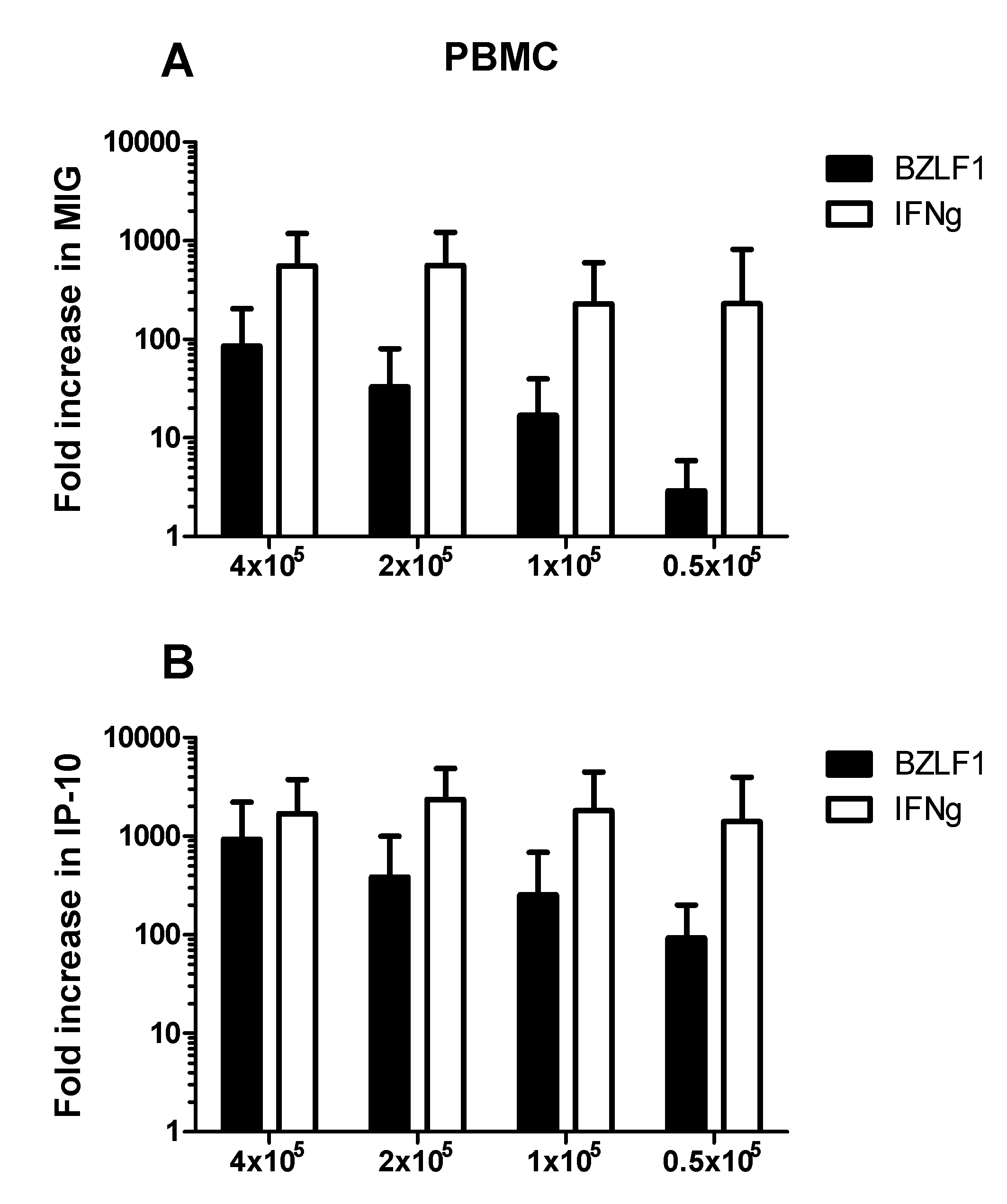

Supplement: Additional file 1 — Detection of antigen-specific responses with reduced numbers of PBMCs. Induction of MIG (A) and IP-10 (B) mRNA in response to stimulation with BZLF1 peptides or recombinant IFNγ in decreasing numbers of PBMCs is shown. (Results are presented as RQ values normalized to the negative control ACTs). Significant induction of MIG and IP-10 can be detected with as few as 1 × 10^5^ cells/well for MIG or 0.5 × 10^5^ cells/well for IP-10. [file 1479-5876-9-143-S1.TIFF]
